# Professor Pancoast’s Clinic, (Surgical)

**Published:** 1844-12-14

**Authors:** Edward R. Squibb


PROFESSOR PANCOAST'S CLINIC, (SURGICAL.)

(Reported by Edward R. Squibb.)

*Saturday, Nov.* 16, 1844.

At the commencement of the lecture of to-day the Professor briefly called the attention of the class to the condition of the patients upon whom he had operated at the last meeting, and firstly to the case of the young gentleman who had been so severe a sufferer from the profuse administration of mercury. This patient had (as will be seen by reference to the previous report, in which the history of the case, and a detail of the first operation are noted,) for 13 years been suffering from a false anchylosis of the jaws, on account of which he was not only totally unable to masticate his food, but could only get it into his mouth in pieces the size of a pea, which he was obliged to introduce through the fissures which existed between his irregularly formed molar teeth and the opening which the sloughing had left in the cheek of the right side. The operation then performed was limited to the overcoming of the resistance constituting the anchylosis, and consisted in the removal of the band of cicatrix and the division of the anterior portion of the right masseter muscle, which was so far successful as to enable us at once to effect an opening of the jaws to nearly the natural extent. By the application from time to time of the jaw dilator of Heister, and the subsequent division of a part of the masseter muscle of the left side, and a separation of the cheek from the gums of that side, which were found preternaturally adherent, the jaws have been separated to the full extent desired, and been kept apart by the interposition of pieces of cork, during the partial cicatrization which has already taken place on the inside of the cheek from which the cicatrix was removed, in so much that the patient, as you see, is now, by the mere exercise of his volition, able to open and shut the mouth. It will yet, however, be requisite to keep up for some time the application of the same measures, in order to overcome the stiffness of the muscles, resulting from so long a period of inaction, as well as to prevent the parts from being tied down anew by the formation of a second cicatrix. The succeeding steps by which the soft parts are to be replaced or restored, must necessarily be postponed for a couple of weeks, although the success of this first step has been quite as full as could have been hoped for under any circumstances.

The lecturer observed that he was desirous of showing the patients upon whom operations have been performed, in order that the gentlemen of the class may not only become familiar with the manner of their performance, and the way in which the first dressings are applied, but also with their success or failure, as well as with the treatment to be instituted under the various occurrences which may arise from unforseen agencies. He would, therefore, beg the gentlemen to give him their attention whilst engaged in this part of his duty, which although seemingly little attractive, would be found, in a practical point of view, to be of the greatest value.

This patient, from whom, as you will remember we last week removed the great toe, is doing very well, the lips of the wound are united nearly throughout by first intention, and he will no doubt in a short time be quite restored to the use of his foot. During this course I shall probably be able to show you, in some cases of greater consequence, the benefits derivable from the appropriate after treatment of operations, in which I place so high value, that to the great attention which I give in this respect, do I mainly attribute the success which I have met with in operations,—a success as great, I believe, as any reasonable man could expect. I have been in the habit of following all operations of any extent, save where there is a constitutional intolerance of the drug, by the soothing and sustaining effects of doses of opium or some of its preparations, repeated at regular intervals for the first two or three days. The benefit which I have seen result from it, seems to arise from its tendency to suppress the irritation, inflammation, and the evil consequences which follow in their train. The dressings, consisting of the adhesive straps and the greased lint, should not in such cases be disturbed until loosened by the establishment of suppuration. By this mode of proceeding, the parts are kept supported in a state of perfect rest during the period at which adhesion or union by first intention takes place, at the same time that the healthy suppuration and granulation in such parts as do not so unite, is not in its commencement disturbed by meddlesome interference with the dressings. After the suppuration has been fairly established, however, which may be known by the discharge of pus, or by the smell which accompanies it, the first dressing should be removed—a daily change afterward is not only useful but necessary to the patient's comfort, in order to bring about a speedy termination of the process in healthy cicatrization. Thus we see the surgeon is but the handmaid of nature, and should in such instances be her handmaid alone, for his active interferencewith these processes always results in their retardation, and the defeat of the object which he has in view. If, however, from some vitiated condition of the economy, or the accidental irritation of the part, so much action should be excited in the stump as to give rise to symptomatic or inflammatory fever, he may be required to interfere, and it is then that his cooling applications to the stump, the internal administration of the grateful neutral mixture, with morphia, tart. of antimony and potassa, low diet, and depletion, may be called for, in order to bring the general functions of the body back again as nearly as possible to the healthy standard.

I next call your attention for a moment to the patient upon whom the operation for excising a carious portion of the os calcis was performed. You will remember that in this case I made a kind of T incision, by which I was enabled to lay bare the bone, and remove the diseased portion with facility. The pieces of charpie or French lint, which were interposed between the lips of the wound, have given free vent to the suppurative discharge which has necessarily followed, with which any remaining diseased fragments of bone may escape, so as to leave the bone in a condition for healthy granulation. This we may expect soon to take place, the flaps will then be brought down with adhesive strips and a roller bandage, and the patient we believe be perfectly cured; having, as he says, suffered little pain from the operation, and being entirely relieved from that gnawing distress of the part which he suffered before.

The next is the patient for whom we opened the tumour in the perineum. This is, in a practical point of view, a very important case,—one to whose diagnosis and treatment you cannot give too much attention; insomuch that you will be likely to encounter many cases of this sort in practice, and if the evil should be suffered to proceed without prompt and active measures for its relief, would inevitably lead to the most disastrous results; namely, extensive and burrowing deep-seated abscess in the perineum, with the distressing and sometimes fatal consequences to which they lead. This patient presented, as was noticed at the time when brought in upon his bed, that worn and broken down appearance, which is almost pathognomonic of these affections of the urinary organs and their appendages. Now, as you see, he walks in with an elastic step, indicating much improvement in his general condition since Saturday last. This of course cannot be fully re-established during the existence of the long standing organic strictures of the urethra, upon which the tumour and abscess depended, and we shall therefore now turn our attention particularly to them. The obstruction which these offer, obliges the patient to discharge his urine almost guttatim, showing to us their extreme narrowness, and the difficulties which we may expect in the introduction of bougies for their dilatation.

I shall now attempt to introduce this elastic bougie more for the purpose of ascertaining the seat and character of the strictures, than with any hope of being able to pass it entirely into the bladder. The instrument, as you see, is of small size, slightly curved and fitted with a stilette. We find the first one existing about three inches from the orifice, through which the instrument by a little manipulation is made to pass. Proceeding then with the greatest care, we now come upon another and exceedingly firm one, as ascertained by the resistance which it offers to the passage of the instrument. Now we should not go to work and by main strength endeavour to force our way through it, even if we are sure, which we cannot always be, that the point is in the right direction; but on the contrary we are by gentle insinuation alone to try to pass the instrument, asking the patient to bend his body forwards, by which the abdominal muscles and suspensory ligament of the penis are relaxed, straitening the canal at the same time by traction on the penis, (which after a time we may again relax) and by slightly changingthe direction of the instrument, so as to allow it to accommodate itself to the canal.

By this course, and by keeping up a slight continued pressure, we find that it has entered the stricture. Now, gentlemen, this is one of the most delicate operations in all surgery, this entering a bougie into a strictured urethra, requiring as much tact and care, as almost any other operation; and very important is it, too, that it should be gently done, for the rough and careless handling of a bougie in endeavouring to effect it, is often, very often followed by rupture of the coats of the urethra, which is made but too readily in the softened and diseased conditions of the lining membrane, at the face of the stricture.

The instrument is now, from the resistance offered to its withdrawal, entered within the stricture, where we shall suffer it to remain for a time, before attempting its farther insertion, in order that by its gradual dilatation of the passage we may be enabled to continue its course into the bladder. I shall, therefore, withdraw the stilette, and place the patient in bed for the present, directing a continuation of the tonic infusion of uva ursi and buchu, with a little bicarbonate of soda, with the object of rendering the urine less acrid and irritating. In the instance of not being able to introduce an ordinary bougie, I had intended to have used one of the small catgut instruments which you see upon the table, which, if once passed into the stricture, would, by its property of absorbing fluid, and becoming enlarged, have dilated the passage so as to admit of a much larger one after its removal. Failing with this, too, we should have tried the wax or plaster bougie of Hunter which is now daily used by Civiale, of Paris, and by many other surgeons both in this country and Europe. It is, as you see, tapering at the point, and is made by rolling waxed cloth between two pieces of marble, until it becomes very firm and hard. It is often very useful from the fact that the point softened by being inserted in warm water, can adapt itself to the narrowness and irregularities of almost any stricture, whilst the body is not so pliable as to prevent the necessary gentle pressure.

I now have two operations to show you, the necessity for which has been occasioned by syphilitic disease. And as there happens to be in the house at this time a pretty full series of the different stages of this affection, I have determined to say something to-day upon the subject, confining myself, however, principally to its first stages, known under the name of chancre and bubo, the progress and treatment of which I will illustrate by cases. The ordinary term syphilis, or lues venerea, is applied to the disease in its second or third stages, or after it has become constitutional; the primary or local affection being designated as chancre. We have thus three stages of syphilis. First, that characterized by the existence of chancre, independent of any other affection. This is the purely local disease, which unchecked is generally quickly followed by symptoms characterizing the second form of the disease of Ricord, or the implication of the first order of parts of Mr. Hunter, viz. of the skin and mucous membranes, the prior determination to either of which tissues in preference to the other, depending upon constitutional peculiarity. Between these two stages we have commonly the bubo marking the progress of the primary toward the secondary stage; upon this second stage, if it be not checked by treatment, commonly succeeds the tertiary form of Ricord, or that stage which implicates the bones and hard tissues of the body, named the second order of parts by Mr. Hunter. Of the origin and first causes of the specific nature of syphilis nothing is known; some chapters in the old Bible rendering its existence probable at the times there spoken of, it being possibly one of the diseases which so afflicted the Jews in their wanderings in the wilderness. It is not improbable but that it may have given rise to their practice of circumcision, which by inducing greater cleanliness, and by thus uncovering the glans, so as to leave no room for the lodgment of the poison under the prepuce, and hardening its lining membrane by exposure, contributed to diminish considerably the risk of infection, which the class of the uncircumcised, whom they hated so much, might have been the means of spreading. We know that the practice is still enforced as a religious rite, among the Turks and Arabs, as well as the Jews. Its first appearance in Europe, however, was about the time of the discovery of America, and hence it was then called the American disease. About the same time, too, it spread to a great extent in the allied French and Spanish army attacking Naples; and therefore received the names of the French disease, the Spanish disease, the Neapolitan disease. At this period its severity was excessive, and its results most destructive and fatal, in comparison to what we see it at the present day. This melioration of the disease is probably due to the advance of civilization, with its inovations upon manners, customs, dress, diet, &c., and most probably in a great extent also to the adoption of more judicious modes of treatment. That the production or propagation of syphilis is as much the effect of a specific virus as small pox, or hydrophobia, has been fully proved by Hunter and Ricord. The inoculation of the matter from a syphilitic sore being just as certain of reproducing the disease as in either of the cases mentioned; and in the inoculation of the disease by natural or artificial means, a very little matter goes a very great way. The experiments lately made by Ricord go to prove, with great clearness, the certainty and constancy with which it occurs, the history and stages being as fully known as those of the small pox or vaccine pustule. At the end of the second day after the introduction under the cuticle, there becomes apparent to the touch a small pimple, sometimes feeling as though a grain of sand had been inserted beneath the skin,—this soon becomes surrounded by a blush, and developes a transparent vesicle, which from containing thin serum is about the fifth day converted into a true pustule, umbilicated at the top like that of small pox. The rupture of this pustule takes place soon after, converting it into an ulcer with a hard base surrounding it, the bottom of which ulcer instead of presenting healthy granulations, has an irregular sloughing appearance, like the torn edge of a piece of pasteboard which has been soaked in water. This last grey appearance, with ragged vertical edges, and a hardened base, constitute the characteristics of the true Hunterian chancre, modified somewhat of course by the situation which it occupies, and some other circumstances which may attend it. The matter from one of these pustules is found to be in a good condition for re-inoculation about the end of the fifth day, at which time it is not generally quite fully formed. Thus we know certainly that if such pustule is thoroughly destroyed by cauterization, before the fourth day, that with it is destroyed all possibility of constitutional symptoms, as it is not before this time that the malignant qualities of the virus are established.

We are very well aware that in some instances it does not require for its propagation, that the virus should be introduced beneath the skin, but that the absorption which takes place from the mucous membrane of the glans, prepuce and urethra, are fully competent to its reproduction. It also frequently happens that a sufficient quantity is introduced beneath the scales of the cuticle upon the body of the penis or the scrotum to produce chancre, as must have happened in one of the cases before us, where, as you see, in addition to those upon the glans and prepuce, we have several large chancres upon the body of the organ.

Chancre is seldom met with by us in the vesicular form; first, because patients do not apply sufficiently early; and again, because the common seat of chancre is upon the glans or prepuce, where the cuticle is so thin that it is ruptured before the vesicle is fully formed; therefore, the condition at which we most frequently see them is, as you see in this case, of small flattened ulcers, with hardened base and ragged vertical edges, which constitutes the simple chancre.

At times, under conditions of great susceptibility, we see the whole penis early thrown by a chancre into a state of excessive irritation, giving rise to the variety called phagadenic chancre, which quickly involves a part or even the whole of the organ in ulceration and sloughing. Such conditions generally arise from a scrofulous cachexia, or from a vitiated condition of the nutritive function, often found dependent upon long sea voyages, or where there is great constitutional irritability. The most terrible death which I ever have witnessed was one occasioned in this manner seventeen years ago, when I was a student in this house. A young black man contracted the disease, which soon exhibited itself in this phagadenic form, and in the course of a few days involved in the slough the whole body and crura of the penis up to their attachment to the pubic bones. The anguish which this patient suffered was almost indescribable. Without the power of sleeping, he kept upon the naked floor, seated upon his hams, like an Arab of the Desert, till death put an end to the terrible scene.

We sometimes, on the other hand, have the ulceration of a very superficial character, appearing to be elevated above the surrounding skin, which accordingly has been named ulcus elevatum. Ulcers of this character appear to be less malignant, granulations being seen in them.

In the case now before us, the ulceration seems to have been of a sloughing character, from the destruction of parts which it has produced, and has occasioned a kind of phymosis, as you see. This is under treatment by nitrate of silver and the black wash, (consisting of calomel and lime water,) and appears to be doing very well—the granulations presenting a healthy appearance.

Cases of syphilis occasionally show themselves, and I here present you with an instance to prove it, where the disease makes its advent, as it were, by a double step, in which the matter seems to have reached the glands through the absorbents, without having produced any chancre, and bubo is in such instances the first expression of the disease.

Another case is here, in which you see the chancres have invaded the prepuce, producing those sores of a chapped or fissured character which are peculiar to mucous membranes subject to corrugation, as those leading into cavities. We have here, also, from the patient's complaining of soreness there, in all probability some chancres upon the glans, which we are prevented from seeing by the phymosis. Those upon the outer surface of the foreskin, according to the patient's statement, have existed for two months.

Now, what is the proper treatment for these chancres? Why the first thing to be done is to give a healthy basis to the ulcer by means of a caustic. This, although it be not in time to insure the patient against the secondary form, is always proper, inasmuch as it lessens the chances of its occurrence; and then we are to excite the parts to a healthy action by dusting it with calomel, or by the use of some slightly stimulating wash, as the black, (consisting of calomel and lime water,) yellow, (consisting of corrosive sublimate and lime water,) or blue, (consisting in a solution of sulph. of copper,) or in later times, as used by Ricord, the aromatic wine, (which is an infusion of some aromatic herbs in wine.) These, in cases of violent inflammation, would of course be inadmissible, and instead, we must use cooling sedative and emollient poultices; purge with blue mass and rhubarb, or some other form of cathartic, and follow up with simple or opiate diaphoretics and warm bath—keeping the patient rigidly, during the time, confined to his bed. In short, we must always modify our treatment in accordance with the nature of the case. The question may arise as to how often the caustic should be applied to a chancre? And to it I would reply, use it boldly and freely until the specific character of the ulcer disappears, and then lay it aside; for I have often seen its prolonged use produce sloughing, which was as hard to overcome as the original chancre would have been; after this you cover the surface of the sore with some mild stimulant, as the aromatic wine, or comp. tinct. of benzoin, with dry calomel, or the gray oxide which precipitates from the black wash, with any of the before named washes, or with what I prefer and generally use, a mixture of calomel, red precipitate, and impure carbonate of zinc, dusted over the surface. This slightly stimulates, at the same time that it protects the granulations, and places them in the best condition to cicatrize. The blue stone, or sulphate of copper, is sometimes used as a caustic, but it will not do to depend upon in the first instance. In some cases, where there is a deep firm slough, or a hardened elevation, and a very strong caustic is required to reach the base of the disease, the Vienna paste or caustic potash may be used; but the one in general use, and sufficient for ordinary cases, is the common nitrate of silver or lunar caustic. In cases like this one before us, where chancres exist upon the glans, whilst there is present a phymosis, which prevents our getting at them with the solid caustic, we must have recourse to the injection of it in solution, or of some of the various washes before noticed, as it will not do to lay open the foreskin whilst invaded by the disease, unless we wish, by the introduction of the virus into the wound, to convert the whole of it into one long chancre.

The next case to which I would direct your attention is one of bubo, which has arisen from a chancrous condition of the glans. This, as you are probably aware, is an enlarged condition of the lymphatic glands of the groin with tumefaction of the surrounding cellular structure, and attributable to the absorption of virus from the penis. Now, it is a curious fact that, whilst these glands are thrown into the condition in which you see them here, by the virus, the absorbents themselves remain unaffected; the first appearing to be like the station houses upon our rail roads, where every thing that comes along must be inspected, and nothing contraband be allowed to pass.

These enlargements, even during the existence of chancre, may be one of two kinds; one produced by the virus transmitted, and the other by sympathetic extension of common inflammation up the tract of the absorbents. But inasmuch as it is not often possible to make a distinction, until the bubo suppurates, and we can test the quality of the matter by inoculation, and as the treatment must be the same in the first stages, this division is less important in respect to practice.

The treatment of bubo must of course be modified by the stage or state in which you find it. If you see the tumour early, before it is yet very large or very sensitive, it is always proper to attempt to discuss it, by the application of some slightly stimulating and discutient plaster. That which I prefer, and with which in simple cases I have rarely failed, is made with equal parts of mercurial and litharge plaster, with a drachm or two of camphor to the ounce, spread upon soft leather and applied over the part—the patient to be put, if possible, to bed, and at least kept quiet until the tumour has entirely disappeared. This last is a very important item in the treatment, insomuch that if I find a patient who will not submit to it, I at once wash my hands of the responsibility in his case; as he will never get well without suppuration, if, by constant motion of his limbs, he keeps up constant irritation in the parts at the bend of the groin. In cases, however, which have gone to the extent which this one has, where we have this blush upon the skin, I would first apply leeches, and then, by lead water and laudanum, or by a mixture of the liq. plumb. subacet. with tr. opii and spirits, and the exhibition of one or two brisk mercurial cathartics, reduce the inflammatory action, which is, as you see, very high. When you get the disease on a backward track, the revellent action of blisters are found often to be useful. Even where some pus has formed below the skin, you may occasionally with blisters stimulate the absorbents to take it away. When they fail in doing this, they often act beneficially in bringing the matter nearer the surface, and lead to an earlier evacuation of the tumour. But where matter forms to much extent, it is always, I believe, the best practice to discharge it at once by a puncture.

In times gone by, mercury was resorted to for the cure of all forms of syphilis, as the one only means by which this end could be attained; and even at the present day it is recommended by some of the highest authorities. The great mass of practitioners with us, however, have discarded its use as a specific, and employ it as they would in any other form of disease, namely, for its alterative and cathartic properties, and not as a specific or antidote to the poison of the disease.

The next case affords you a striking example of what are called mucous tubercles; an affection which appears to hold a middle place between the malignancy of syphilis and the ordinary benign affections of the skin. They generally appear upon the nates, in the axilla, or in the mouth, as is exhibited in the present case. They cannot be propagated by inoculation, but may be communicated by coition; being for the most part a mild affection, and not very difficult to treat. I have seen much worse cases than this, some in which they stood out around the anus like the comb of a cock, or a cauliflower, and yet they readily yielded to washing with a strong solution of chlor. of sodium, and a dusting of the parts with some slightly stimulant powder—the one which I have used most consisting of two parts of calomel and one of red precipitate.

Here I present you with a case upon which I will now perform the operation of circumcision, removing by this means a crop of recent chancres upon the end of the prepuce, which, as you see, is very long and contracted at its extremity, so as to leave scarcely an outlet for the urine, forming a phymosis, which threatens to give the patient some inconvenience. There being here no disease of the glans, and the chancres being of recent occurrence, we shall run little risk in the operation, which will at some period have to be done, and probably rid the patient of two evils at once, and at all events protect him against the development of chancres on the concealed glans. The operation is a very simple one, consisting in grasping the skin between the handles of a pair of dressing forceps, taking care not to grasp the glans, and then by a single cut remove the part. Inasmuch as the skin is attached to the penis nearer to the end below than above, it is necessary to grasp it obliquely, and also to be careful that no more than a just portion lies outside the forceps; as I have seen the operation performed where half of the skin of the penis was removed by drawing it forwards between the forceps. It is necessary, as a second step, sometimes to trim off the mucous membrane, which is generally left uncovered by the retraction of the skin; and in this case I shall do what I do not often deem necessary, namely, close the two together by interrupted sutures, so as to get, if possible, immediate union, and diminish the risk of having the raw surface affected by the poison. I shall, perhaps, have to show you during the winter an operation for phymosis, which is peculiar to myself, the advantages of which I shall then explain.

The other operation which I have to show you to-day is that of amputation of the penis. This is an operation very seldom resorted to, and then as is almost always the case, for cancerous affections of the organ. This is the patient exhibited to you at our last meeting, who had infiltration of urine into the corpus cavernosum which could be squeezed out through the ulcerated openings on the dorsum as from a sponge, the anterior part of the urethra being totally obliterated. Now the benefit which is to arise from this operation, is principally that of affording a more free passage to the urine at the same time that we remove the constant pressure and tendency to sloughing; allowing the urine to take a more direct course, and make for itself a new passage. As the glans, the sensitive part of the organ is totally destroyed, one most serious objection to the proceeding is removed.

Operation.—I place as you see, an assistant behind the patient to make compression at the root of the penis to prevent my taking off too much of the skin, and then by a single oblique cut with a catling downwards, remove the end of the stump. We find here less bleeding than we expected, that little coming from the dorsal artery of the penis; we find the whole cellular structure of the corpus cavernosum degenerated and blended into a condensed mass, through which as you see, the urine escapes in small streams; one of these several orifices will now probably enlarge and establish a passage for the urine. There appears to be no vessels to be tied at present, but if bleeding should take place, they may be tied by very delicate ligatures. The application of a little lint and a bandage being all the dressing that is necessary, I shall not take up your time with their arrangement, in as much as the period allotted to me here, has now expired.

